# Exploring the Relationship Between Childhood Maltreatment, Alexithymia, and Facial Emotional Recognition in Schizophrenia

**DOI:** 10.1002/brb3.70752

**Published:** 2025-08-12

**Authors:** Cong Wang, Tongbao Zhan, Jun Zheng, Huanshun Hu, Mingming Zheng, Lei Dai, Weiwei Tong, Xulai Zhang

**Affiliations:** ^1^ Huainan Mental Health Center Huainan China; ^2^ The Fourth People's Hospital of Hefei Hefei China

**Keywords:** alexithymia, childhood maltreatment, facial emotional recognition, schizophrenia

## Abstract

**Background**: Childhood maltreatment is a significant factor affecting social cognition in schizophrenia (SCZ) patients. However, the relationship between childhood maltreatment, alexithymia, and facial emotional recognition in SCZ remains unclear.

**Methods**: SCZ patients in stable phase (*n* = 90) and healthy controls (*n* = 47) were included according to the DSM‐5 criteria. Clinical symptoms were assessed using the Positive and Negative Syndrome Scale (PANSS), childhood maltreatment was evaluated using the Childhood Trauma Questionnaire (CTQ), and alexithymia was assessed using the Toronto Alexithymia Scale‐20 (TAS‐20) to evaluate the ability to identify, describe, and express emotions. Social cognition was assessed using the Facial Emotion Recognition Test.

**Results**: Our findings indicate significant differences in CTQ, TAS‐20, and facial emotional recognition between the two groups, with the SCZ group showing more severe impairments. Pearson correlation analysis showed that correct facial emotion recognition was negatively correlated with childhood maltreatment and alexithymia (*p* < 0.05). Stepwise regression analysis further revealed that the total PANSS score, positive symptom, CTQ total score, and difficulty describing feelings negatively affected the accuracy of facial emotional recognition (*p* < 0.05). Patients who find it difficult to describe feelings may also have greater difficulty in recognizing facial expressions of anger.

**Conclusion**: Good psychosocial functioning can mitigate the negative impact of childhood maltreatment on the severity of illness in SCZ patients. Therefore, psychotherapy that promotes personal expression is useful in the treatment of SCZ.

## Graphical abstract

The study investigates the relationship between childhood maltreatment, alexithymia, and facial emotional recognition in patients with schizophrenia. The results indicate that childhood maltreatment and alexithymia negatively affect facial emotion recognition in patients with schizophrenia, especially their ability to recognize anger. Improving emotional expression abilities may help alleviate the negative effects of childhood maltreatment on the patients' social cognitive functions.

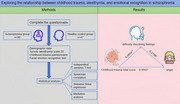

## 1 Introduction

Schizophrenia (SCZ) is a serious mental disorder with a high incidence of morbidity and disability (Harris 2023; Jauhar et al. 2022). Patients often exhibit significant impairments in multiple areas of cognitive function, such as attention, memory, executive function, and social cognition (Gebreegziabhere et al. 2022). Presently, second‐generation antipsychotic (SGA) drugs, which are effective in relieving positive symptoms of the disease and have a low propensity to cause extrapyramidal side effects, became the mainstay of SCZ treatment (Divac et al. 2014). However, cognitive function in patients with SCZ does not improve comprehensively or completely.

It has been reported that social cognition has a stronger association with functional outcomes and is considered a higher‐level cognitive ability (Bell et al. 2009; Fett et al. 2011). Social cognition primarily includes empathy, alexithymia, and facial emotion recognition, which involve the understanding and interpretation of others’ emotional states and social signals (Di Tella et al. 2020). Alexithymia refers to difficulties in recognizing, expressing, and understanding one's own and others’ emotions (Haviland et al. 2004). Social cognitive abnormalities, such as alexithymia, can exacerbate symptoms and severely affect the health and quality of life of patients (Huo et al. 2023). Numerous studies have shown that patients with SCZ often have high levels of alexithymia (Xiao et al. 2024; Yi et al. 2023). Previous studies have found that the sudden outbursts of anger observed in patients with violent SCZ may represent inappropriate responses to emotional stimuli, potentially related to impaired perception of others’ facial emotions (Sedat et al. 2013; Sedgwick et al. 2017). Therefore, accurate recognition and expression of emotions are crucial for the establishment, maintenance, and adaptation of interpersonal relationships.

Adverse childhood experiences have been identified as significant risk factors for the development of various psychiatric disorders, including SCZ, bipolar disorder, borderline personality disorder, major depressive disorder, and post‐traumatic stress disorder (Aas et al. 2016; Cattane et al. 2017; Maercker et al. 2004; Read et al. 2001). Childhood maltreatment is mainly categorized into five subtypes: emotional abuse, emotional neglect, physical abuse, physical neglect, and sexual abuse (Cruz 2023). A meta‐analysis revealed that higher levels of childhood maltreatment exposure are associated with increased levels of alexithymia in adulthood (Ditzer et al. 2023). In addition, evidence from neuroimaging studies has shown that individuals with a history of childhood maltreatment exhibit heightened amygdala responses to facial emotions in adulthood, particularly to faces perceived as threatening (Aas et al. 2017; Cancel et al. 2017). For example, childhood maltreatment patients have an increased fear bias, tending to misinterpret expressions of sadness, surprise, or anger as expressions of fear (Suzuki et al. 2015). Functional magnetic resonance imaging studies have found that patients with a history of childhood maltreatment process emotions with reduced connectivity between the amygdala and the occipital face area—a brain region associated with facial recognition—which may impair the processing of facial expressions (Huang et al. 2024).

**FIGURE 1:**
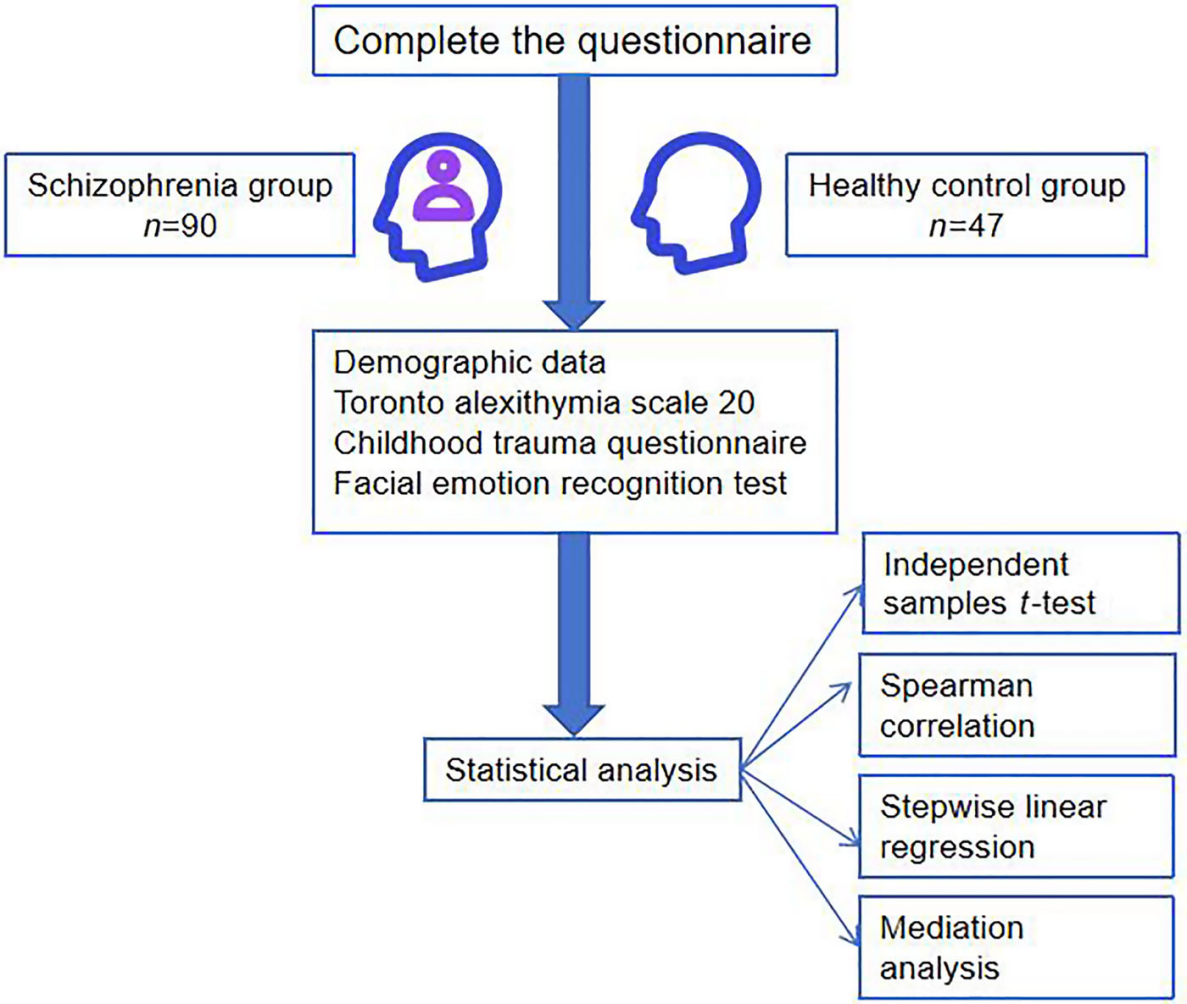
Flowchart.

**FIGURE 2:**
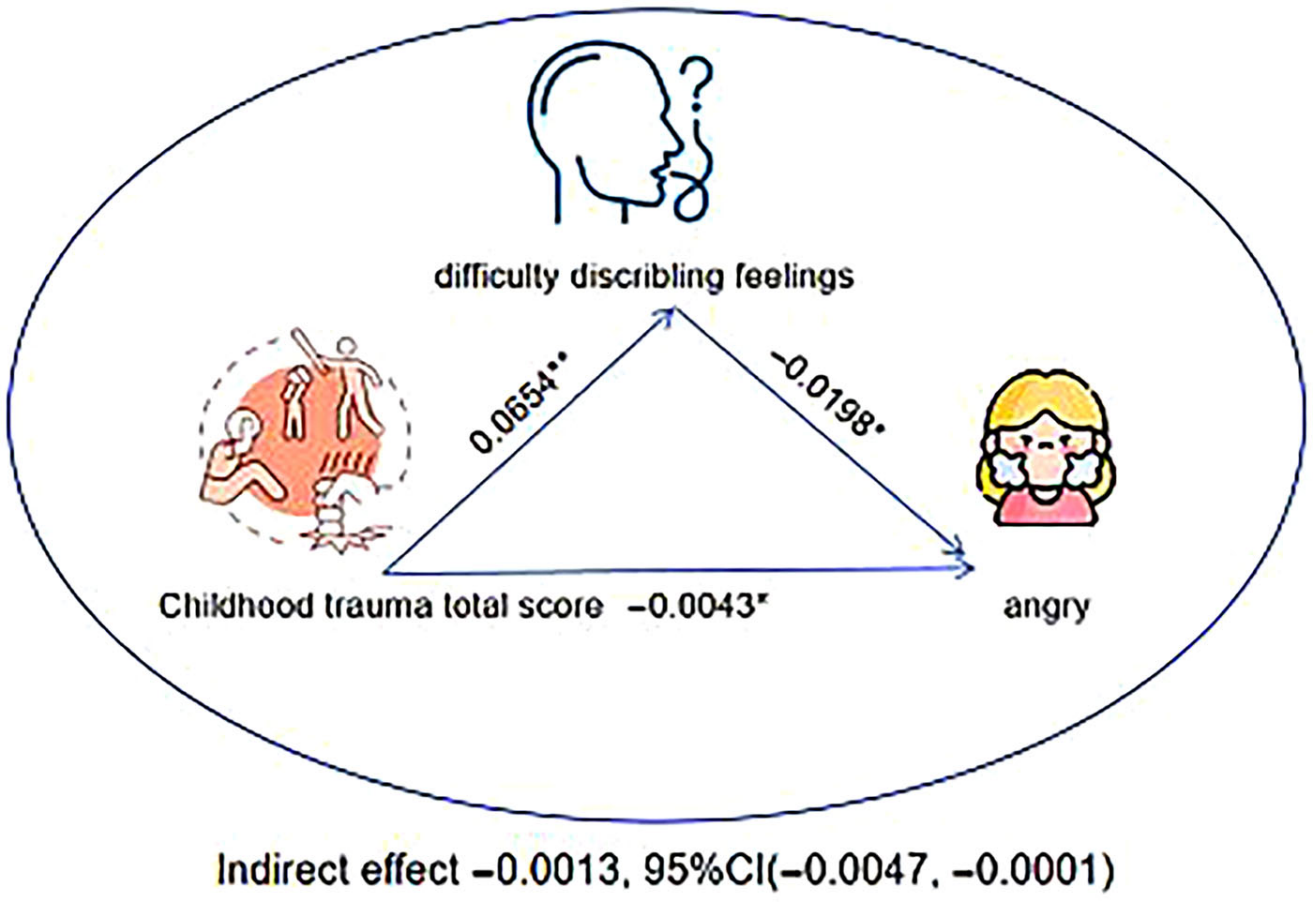
Mediation analysis of the role of difficulty describing feelings in mediating the relationship between childhood trauma and emotion recognition. CTQ, Childhood Trauma Questionnaire; ^*^ *p* ≤ 0.05; ^**^ *p* ≤ 0.01.

In light of this, from the perspectives of developmental psychology and psychopathology, the present study aims to explore the associations among childhood abuse, alexithymia, and facial emotion recognition, in an effort to understand the mechanisms underlying social cognitive impairments from the perspective of longitudinal psychological development. To minimize the influence of cultural and sociological factors, only participants of Han Chinese ethnicity were included in the study (Zhang et al. 2021). The hypotheses of this study are as follows. First, compared to healthy controls, the patients of SCZ are more impaired on the Childhood Trauma Questionnaire (CTQ), the Toronto Alexithymia Scale‐20 (TAS‐20), and the Facial Emotion Recognition Test. Second, facial emotion recognition in SCZ patients is expected to be significantly correlated with childhood maltreatment and alexithymia. Third, we will explore whether the relationship between childhood maltreatment and facial emotion recognition in SCZ is mediated by alexithymia Figure 1, 2.

## 2 Material and Methods

### 2.1 Participants

This cross‐sectional cohort research was conducted at Huainan Mental Health Hospital between October 2024 to December 2024.

Two senior psychiatrists used the Mini‐International Neuropsychiatric Interview (MINI) 6.0.0 to confirm the psychiatric diagnosis of all participants; in addition, two trained psychiatric residents independently scored for each participant with the related scales, the mean scores taken by them were used as the final score.

Inclusion criteria: (1) Diagnosis of SCZ according to the Diagnostic and Statistical Manual of Mental Disorders, Fifth Edition (DSM‐5) and re‐diagnosis of mental disorders using the MINI 6.0.0, (2) age between 18 and 60 years, and (3) with no history of traumatic brain injury or any neurological disease.

A total of 90 patients meeting the inclusion criteria and not the exclusion criteria were included.

Individuals in the healthy group met the following inclusion criteria: (1) Aged between 18 and 60 years and (2) healthy volunteers were assessed with MINI 6.0.0 to rule out any psychiatric conditions. All the participants did not have any of the following exclusion criteria: (1) Diagnosis of other psychiatric disorders, (2) history of neurological disorders or head trauma, (3) history of alcohol or substance abuse, (4) history of electroconvulsive therapy or transcranial magnetic stimulation within the last 6 months, and (5) pregnant or breastfeeding women.

Finally, 47 healthy people were recruited from the local community into the healthy control group (Figure 1).

This study was approved by the Medical Research Ethics Committee of Huainan Mental Health Hospital, approval number: HNSYKJ2024001. In this study, all participants signed an informed consent form prior to assessment.

### 2.2 Assessments MINI 6.0.0

MINI 6.0.0, which is a brief and straightforward diagnostic interview for psychiatric disorders used by psychiatrists in the United States and Europe, was used to confirm the preliminary clinical diagnosis. The reliability inter‐rater and test–retest of MINI 6.0.0 were excellent and its kappa values were above 0.80 and 0.90, respectively. All patients underwent MINI to verify the clinical diagnoses of first‐episode SCZ patients (Kadri et al. 2005).

### 2.3 Positive and Negative Syndrome Scale

The Positive and Negative Syndrome Scale (PANSS) was used to assess the severity of symptoms in SCZ patients. The scale includes 30 items, each rated based on the severity of the symptoms, with scores ranging from 1 (*no symptoms*) to 7 (*severe symptoms*). In this study, the Cronbach's *α* coefficient for this test was 0.92 (Wu et al. 2015; Yehya et al. 2016).

### 2.4 Childhood Trauma Questionnaire

The CTQ is an instrument designed to explore childhood abuse history. It consists of 28 items, of which 25 are grouped into five subscales: emotional abuse, physical abuse, sexual abuse, emotional neglect, and physical neglect. The remaining items constitute a validity scale (items 10, 16, and 22). Each item is rated on a 5‐point Likert‐type scale from 1 (*never*) to 5 (*almost always*). There is a total score and independent scores for the five subscales. Therefore, the total score ranges from 25 to 125 and the subscale scores from 5 to 25. In this study, the Cronbach's *α* coefficient for this test was 0.81 (Jiang et al. 2018).

### 2.5 Toronto Alexithymia Scale‐20

The TAS‐20 was used to assess individuals’ ability to identify, describe, and express their own emotions. The TAS‐20 consists of 20 items, each rated on a 1–5 scale, and is divided into three subscales: difficulty identifying feelings, difficulty describing feelings, and externally oriented thinking. In this study, the Cronbach's *α* coefficient for this test was 0.83 (Joukamaa et al. 2001).

### 2.6 Facial Emotion Recognition Test

The Facial Emotion Recognition Test used photographs from the Chinese Facial Expression Image Database, which included facial expressions of happiness, fear, anger, sadness, surprise, and calmness (Gong et al. 2011). The computer randomly presented 48 mixed photographs, including eight images for each emotion. Above each photo, six emotional options were displayed. Participants were asked to select an emotion that matched the displayed facial expression, and the computer recorded the response time and accuracy. The accuracy for each emotion type and the correct recognition count for all 48 facial expression photos were used for analysis.

### 2.7 Statistical Analysis

The *t*‐tests and chi‐squared tests were conducted to compare differences in continuous and categorical variables, respectively, between the two groups. Continuous variables with non‐normal distributions were compared between groups using the Mann–Whitney *U* test and are presented as median and interquartile range (M [IQR]). We used Spearman correlation coefficients with false discovery rate (FDR) judgment to construct the relationship between scales of CTQ, TAS‐20, and facial emotion recognition. Two‐sided tests were conducted with a significance level of *α* = 0.05. Stepwise regression analyses were used to explore the relationship between facial emotion recognition and childhood maltreatment and alexithymia. Finally, the mediation analysis was conducted to further test whether the relationship between childhood maltreatment risk factors and facial emotion recognition was mediated by alexithymia. This analysis was carried out by quantifying the direct and indirect relationships among the independent variables, mediator (i.e., alexithymia), and dependent variable (i.e., emotion recognition). In the mediation models, all paths were reported as unstandardized ordinary least squares regression coefficients. A significance analysis was based on 5000 bootstrap realizations, and a significant indirect effect was indicated when the bootstrap 95% confidence interval (CI) was not zero. All statistical tests were two‐tailed, and statistical significance was set as *α* < 0.05.

## 3 Results

### 3.1 Characteristic Results of Demographics, Childhood Maltreatment, Alexithymia, and Facial Emotional Recognition Between Two Groups

Sociodemographic and clinical characteristics of participants classified into categories are shown in Tables 1 and 2. The results showed no statistically significant differences between the two groups in demographic characteristics such as age, gender, years of education, marital status, occupation, living arrangement, per capita monthly income (*p* > 0.05).

**TABLE 1:** Comparison of sociodemographic characteristics between two groups.

Factors		Healthy group (*n* = 47)	Schizophrenia group (*n* = 90)	*χ*2*/t*	*p*
Age (years)		33.17 ± 11.01	37.18 ± 11.38	1923	0.057
Sex	Male	22 (52.4%)	43 (44.8%)	0.675	0.411
	Female	20 (47.6%)	53 (55.2%)		
Years of education (years)	≤ 9	13 (31%)	45 (46.9%)	3.04	0.081
	> 9	29 (69%)	51 (53.1%)		
Employment status	No	14 (33.3%)	49 (51%)	3.693	0.055
	Yes	28 (66.7%)	47 (49%)		
Marital status	No	20 (47.6%)	62 (64.6%)	3.487	0.062
	Yes	22 (52.4%)	34 (35.4%)		
Live with family	No	16 (38.1%)	22 (22.9%)	3.373	0.066
	Yes	26 (61.9%)	74 (77.1%)		
Monthly income per capita	< 5000	81(90%)	41(87.2%)	0.242	0.623
	≥ 5000	9(10%)	6(12.8%)		

**TABLE 2:** Comparison of childhood maltreatment, alexithymia, and facial emotional recognition between two groups.

Factors	Schizophrenia group (*n* = 90)	Healthy group (*n* = 47)	*T*	*p*
Sexual abuse	6.50 (5.00)	5.00 (0.00)	2982.5	< 0.001
Physical abuse	6.00 (5.00)	5.00 (0.56)	3012.5	< 0.001
Emotional abuse	8.50 (6.00)	6.00 (2.00)	3026	< 0.001
Physical neglect	12.00 (3.75)	10.00 (2.00)	3348.5	< 0.001
Emotional neglect	15.00 (4.75)	13.00 (3.00)	2824	< 0.01
CTQ total score	48.00 (17.00)	39.00 (5.00)	3621.5	< 0.001
TAS‐20 total score	58.00 (14.00)	48.80 (9.00)	3148.5	< 0.001
Difficulty identifying feelings	20.00 (8.75)	14.66 (6.00)	3110	< 0.001
Difficulty describing feelings	15.00 (4.00)	12.00 (4.00)	3103	< 0.001
Externally oriented thinking	23.00 (3.75)	22.36 (4.00)	2467	0.108
Disgust	0.28 (0.25)	0.63 (0.50)	1088	< 0.001
Calm	0.88 (0.25)	0.88 (0.12)	1418	< 0.01
Fear	0.33 (0.37)	0.38 (0.50)	1767.5	0.111
Sadness	0.63 (0.50)	0.88 (0.19)	1432.5	< 0.01
Surprised	0.63 (0.37)	0.75 (0.13)	1269	< 0.001
Anger	0.38 (0.50)	0.63 (0.37)	1438.5	< 0.01
Happiness	0.88 (0.48)	1.00 (0.12)	1405.5	< 0.001

*Note*: Data presented as M [IQR].

Abbreviations: CTQ, Childhood Trauma Questionnaire; TAS‐20, Toronto Alexithymia Scale‐20.

The SCZ group scored significantly higher than the healthy control group on the CTQ total score, and subscales for sexual abuse, emotional abuse, emotional neglect, physical abuse, and physical neglect. Results of TAS‐20 assessment indicated that the SCZ group showed significantly impaired emotional identification, emotional description, and TAS‐20 total score compared to the control group. Likewise, the SCZ group exhibited significantly lower accuracy in recognizing facial expressions of disgust, sadness, surprise, anger, and happiness.

### 3.2 Factors Correlated Between Facial Emotion Recognition With Childhood Maltreatment, Alexithymia

To assess potential risk factors, we used Spearman correlation analyses to assess the relationship between facial emotion recognition and childhood maltreatment, alexithymia; the results are shown in Table 3. First, we found the main negative effect of the five dimensions of childhood maltreatment and the two dimensions of alexithymia on facial emotion recognition correctness. Disgust recognition accuracy was negatively correlated with sexual abuse, physical abuse, emotional abuse, physical neglect, emotional neglect, CTQ total score, and difficulty describing feelings. Calm recognition accuracy was negatively correlated with sexual abuse, physical abuse, emotional abuse, physical neglect, emotional neglect, CTQ total score, TAS‐20 total score, difficulty identifying feelings, and difficulty describing feelings. Sadness recognition accuracy was negatively correlated with sexual abuse, physical abuse, emotional abuse, physical neglect, emotional neglect, CTQ total score, TAS‐20 total score, difficulty identifying feelings, and difficulty describing feelings. Surprise recognition accuracy was negatively correlated with sexual abuse, physical abuse, emotional abuse, physical neglect, CTQ total score, TAS‐20 total score, difficulty identifying feelings, and difficulty describing feelings. Anger recognition accuracy was negatively correlated with sexual abuse, physical abuse, emotional abuse, physical neglect, emotional neglect, CTQ total score, TAS‐20 total score, difficulty identifying feelings, and difficulty describing feelings. Happiness recognition accuracy was negatively correlated with sexual abuse, physical abuse, emotional abuse, physical neglect, emotional neglect, CTQ total score, TAS‐20 total score, and difficulty identifying feelings.

**TABLE 3:** Correlation analysis between childhood maltreatment, alexithymia, and facial emotion recognition.

	Disgust		Calm		Sadness		Surprised		Anger		Happiness	
	*r*	*p*	*r*	*p*	*r*	*p*	*r*	*p*	*r*	*p*	*r*	*p*
CTQ total score	−0.355^**^	< 0.001	−0.397^**^	< 0.001	−0.389^**^	< 0.001	−0.406^**^	< 0.001	−0.376^**^	< 0.001	−0.334^**^	< 0.001
Sexual abuse	−0.250^*^	0.007	−0.300^**^	< 0.001	−0.232^*^	0.013	−0.336^**^	< 0.001	−0.278^*^	0.003	−0.263^*^	0.006
Physical abuse	−0.261^*^	0.006	−0.349^**^	< 0.001	−0.267^*^	0.006	−0.382^**^	< 0.001	−0.249^*^	0.007	−0.311^**^	< 0.001
Emotional abuse	−0.214^*^	0.023	−0.333^**^	< 0.001	−0.204^*^	0.031	−0.296^**^	< 0.001	−0.202^*^	0.032	−0.198^*^	0.035
Physical neglect	−0.214^*^	0.023	−0.250^*^	0.007	−0.217^*^	0.022	−0.230^*^	0.014	−0.246^*^	0.009	−0.190^*^	0.045
Emotional neglect	−0.283^*^	0.003	−0.187^*^	0.048	−0.251^*^	0.007	−0.180	0.057	−0.245^*^	0.009	−0.229^*^	0.014
TAS‐20 total score	−0.186	0.050	−0.355^**^	< 0.001	−0.357^**^	< 0.001	−0.349^**^	< 0.001	−0.321^**^	< 0.001	−0.255^*^	0.007
Difficulty identifying feelings	−0.184	0.052	−0.349^**^	< 0.001	−0.303^**^	< 0.001	−0.354^**^	< 0.001	−0.308^**^	< 0.001	−0.264^*^	0.006
Difficulty describing feelings	−0.211^*^	0.024	−0.301^**^	< 0.001	−0.314^**^	< 0.001	−0.339^**^	< 0.001	−0.331^**^	< 0.001	−0.183	0.053

Abbreviations: CTQ, Childhood Trauma Questionnaire; TAS‐20, Toronto Alexithymia Scale‐20.

^*^ *p* ≤ 0.05; ^**^ *p* ≤ 0.001.

### 3.3 Findings on the Stepwise Regression Model

The stepwise regression model is shown in Table 4. Stepwise regression analyses of childhood maltreatment, psychotic symptoms, and alexithymia were performed to predict risk factors for facial emotion recognition. The final model of the forward regression showed that the PANSS scale total score was significantly negatively correlated with disgust correctness, and that the PANSS scale total score significantly explained 4.1% of the variance accounted for by the variable. CTQ total score was significantly negatively correlated with surprise correctness, significantly explaining 12.7% of the variance accounted for by the variable. CTQ total score, difficulty describing feelings, and positive symptom were significantly negatively correlated with anger correctness, significantly explaining 14.7% of the variance accounted for by the variables.

**TABLE 4:** Stepwise linear regression analysis of emotional recognition.

Dependent variable	Independent variables	*B*	*SE*	*Beta (β)*	*T*	*p*	*R* ^2^
Disgust	PANSS total score	−0.003	0.001	−0.228	−2.197	0.031	0.041
Surprised	CTQ total score	−0.008	0.002	−0.370	−3.740	< 0.001	0.127
Anger	CTQ total score	−0.005	0.002	−0.237	−2.167	0.033	0.147
	Difficulty describing feelings	−0.02	0.009	−0.273	−2.281	0.025	
	Positive symptoms	0.018	0.009	0.2	1.996	0.049	

Abbreviations: *B*, unstandardized coefficient; *Beta*, standardized coefficient; CTQ, Childhood Trauma Questionnaire; PANSS, Positive and Negative Syndrome Scale; *R* ^2^: Adjusted *R* square; *SE*, standard error.

### 3.4 Mediation Between Childhood Maltreatment and Facial Emotion Recognition by Alexithymia

Mediation pathway analysis revealed that difficulty describing feelings played a significant indirect role in the relationship between childhood maltreatment and anger emotion recognition in SCZ patients. The results showed a significant indirect effect (*p* < 0.05), confirming that childhood maltreatment and emotional description ability had a significant mediating effect on anger emotion recognition in SCZ patients (Figure 2).

## 4 Discussion

The main aim of the present study was to investigate the relationship between childhood maltreatment, alexithymia, and facial emotion recognition ability in patients with SCZ. The results demonstrated that emotional description ability plays a significant mediating role in the effect of childhood maltreatment on anger emotion recognition in patients with SCZ, which provides new theoretical perspectives for understanding emotion processing mechanisms and valuable insights for clinical interventions.

A cross‐sectional study of emotional recognition patterns in patients with SCZ found that patients tend to mistake happy faces for negative emotions and often confuse negative emotions, such as fear for anger, which may affect their social interaction (Lee et al. 2022). Early experiences of abuse or neglect may have long‐term effects on patients’ emotion recognition abilities by altering the neural mechanisms involved in emotional processing, particularly the functioning of the prefrontal cortex and the amygdala (Dannlowski et al. 2013). By enhancing the ability to recognize emotions, it may be possible to improve the social functioning of patients with SCZ, thereby reducing the severity of their condition (Wells et al. 2020). Emotional neglect and difficulties in emotional regulation may exacerbate an individual's challenges in recognizing others’ emotions, particularly in identifying negative emotions (Liu et al. 2016; Rokita et al. 2018). Consistent with previous findings, the results of the present study suggest that childhood abuse may negatively affect emotion recognition abilities in individuals with SCZ. Specifically, higher levels of childhood abuse were associated with longer response times and reduced accuracy in facial emotion recognition.

Stepwise regression analysis indicated a negative correlation between cognitive symptoms and excitement‐hostility symptoms in the PANSS and facial emotion recognition ability. These cognitive deficits may make it difficult for patients to process emotional information (e.g., attention deficits, working memory impairments), which could further exacerbate facial emotion recognition difficulties by impairing the emotional processing process, especially in accurately interpreting and responding to emotions (Dannlowski et al. 2013). Meanwhile, cognitive symptoms in PANSS and emotional neglect in CTQ are also significantly related to emotion recognition ability. This is consistent with existing research, indicating that cognitive symptoms and emotional neglect not only affect the accuracy of facial emotion recognition but may also exacerbate social avoidance and limited social functioning (Javed and Charles 2018). Therefore, in clinical treatment, in addition to addressing the core symptoms of SCZ, improving patients’ facial emotion recognition and social cognitive function, especially in the areas of cognitive symptoms and emotional neglect, may help enhance their social adaptability and quality of life.

Previous studies have proposed that the lack of ability to describe emotions may lead to an individual's inability to accurately interpret and respond to the emotional expression of others, thus having a profound impact on his social cognitive function (Montebarocci et al. 2011). Anger, as a highly conflictual and intense emotion, may require greater emotional regulation and social cognitive abilities in social interactions (Weiblen et al. 2021). A study on SCZ patients with a history of violent behavior found that childhood maltreatment may increase the risk of violence by affecting social cognition (Vaskinn et al. 2021). From a psychological perspective, childhood maltreatment may lead individuals to develop a perception that the world is dangerous, resulting in a tendency to misinterpret neutral or ambiguous facial expressions as hostile—such as perceiving neutral faces as expressions of anger (Pollak et al. 2009). A cross‐sectional study found that depressed patients with a history of childhood maltreatment exhibited reduced attention to negative facial expressions, such as anger, which may result in the neglect of critical emotional cues (Bodenschatz et al. 2019). de Bles et al. pointed out that adverse childhood experiences may lead to a reduced sensitivity to emotions, which is manifested in poor recognition of anger expressions in facial emotion recognition (de Bles et al. 2023). This psychological mechanism is also supported by neurobiological evidence. Preclinical studies have shown that early‐life maltreatment may induce long‐lasting alterations in neuroplasticity (Zeng et al. 2020). Experiences of maltreatment may lead to abnormal cortisol levels, which, through glucocorticoid receptor mechanisms—such as FKBP5 gene methylation—can affect hippocampal development (Zhang et al. 2013). Childhood maltreatment may lead to hyperactivation of the amygdala and heightened sensitivity to threat‐related facial expressions (e.g., fear) (Dannlowski et al. 2012; McCrory et al. 2013; Tottenham et al. 2011). In addition, reduced functional connectivity among the hippocampus, anterior cingulate cortex, and ventromedial prefrontal cortex may impair the ability to inhibit exaggerated interpretations of negative emotional stimuli (Hart et al. 2018). Our study, through mediation pathway analysis, found that emotional description ability has a significant indirect effect on the relationship between childhood maltreatment and anger emotion recognition ability in SCZ patients. This finding reveals the key role of alexithymia in facial emotion recognition, as individuals with limited ability to express emotions may face further difficulties in processing complex emotions, especially in recognizing anger. This may suggest that alexithymia serves as a key mechanism linking early childhood abuse to impairments in adult facial emotion recognition, offering a novel perspective on how early adversity shapes the cognitive‐emotional system.

The findings of this study may provide valuable insights for timely interventions in patients with SCZ. For high‐risk children and adolescents—such as those exposed to abuse or neglect—interventions including emotion labeling training and body‐sensation‐emotion integration exercises may enhance emotional awareness and expression, thereby interrupting the negative developmental trajectory. For adults with facial emotion recognition deficits, assessing histories of childhood abuse and levels of alexithymia may help inform the development of personalized treatment plans. Such plans may include trauma‐focused therapy combined with emotion regulation skills training, which could support better emotional understanding and expression, ultimately improving social cognition and psychosocial functioning.

Several limitations of the current findings should be considered in this context. First, the main limitation of the present study is the relatively small sample size. In future research, we plan to continue data collection to increase the sample size and improve statistical power. Second, this study is cross‐sectional research, although our study provides evidence for emotional descriptive ability as a mediating variable, more longitudinal studies are needed to verify causation. Finally, future research should also explore the mechanisms by which other potential mediating variables, such as emotional regulation or social support, function between childhood maltreatment and emotional recognition.

In conclusion, this study reveals the negative effects of childhood maltreatment and alexithymia on facial emotion recognition in patients with SCZ, especially in the recognition of anger. Emotional description ability played a significant mediating role in this relationship, suggesting that improving emotional expression ability may help alleviate the negative effects of childhood maltreatment on patients’ social cognitive function. Therefore, clinical treatment should focus on improving the emotional description ability of SCZ patients in order to improve their social function and disease prognosis.

## Author Contributions

**Cong Wang**: investigation, writing – original draft, data curation. **Tongbao Zhan**: project administration, data curation. **Jun Zheng**: formal analysis. **Huanshun Hu**: formal analysis. **Mingming Zheng**: data curation, funding acquisition. **Lei Dai**: investigation. **Weiwei Tong**: investigation. **Xulai Zhang**: project administration, supervision.

## Conflicts of Interest

The authors declare no conflicts of interest.

## Peer Review

The peer review history for this article is available at https://publons.com/publon/10.1002/brb3.70752

## Data Availability

The data that support the findings of this study are available on request from the corresponding author. The data are not publicly available due to privacy or ethical restrictions.

## References

[brb370752-bib-0001] Aas, M. , C. Henry , O. A. Andreassen , F. Bellivier , I. Melle , and B. Etain . 2016. “The Role of Childhood Trauma in Bipolar Disorders.” International Journal of Bipolar Disorders 4, no. 1: 2. 10.1186/s40345-015-0042-0. 26763504 PMC4712184

[brb370752-bib-0002] Aas, M. , K. Kauppi , C. L. Brandt , et al. 2017. “Childhood Trauma Is Associated With Increased Brain Responses to Emotionally Negative as Compared With Positive Faces in Patients With Psychotic Disorders.” Psychological Medicine 47, no. 4: 669–679. 10.1017/s0033291716002762. 27834153

[brb370752-bib-0003] Bell, M. , H. W. Tsang , T. C. Greig , and G. J. Bryson . 2009. “Neurocognition, Social Cognition, Perceived Social Discomfort, and Vocational Outcomes in Schizophrenia.” Schizophrenia Bulletin 35, no. 4: 738–747. 10.1093/schbul/sbm169. 18245058 PMC2696363

[brb370752-bib-0004] Bodenschatz, C. M. , M. Skopinceva , T. Ruß , and T. Suslow . 2019. “Attentional Bias and Childhood Maltreatment in Clinical Depression—An Eye‐Tracking Study.” Journal of Psychiatric Research 112: 83–88. 10.1016/j.jpsychires.2019.02.025. 30870713

[brb370752-bib-0005] Cancel, A. , M. Comte , C. Boutet , et al. 2017. “Childhood Trauma and Emotional Processing Circuits in Schizophrenia: A Functional Connectivity Study.” Schizophrenia Research 184: 69–72. 10.1016/j.schres.2016.12.003. 27979699

[brb370752-bib-0006] Cattane, N. , R. Rossi , M. Lanfredi , and A. Cattaneo . 2017. “Borderline Personality Disorder and Childhood Trauma: Exploring the Affected Biological Systems and Mechanisms.” BMC Psychiatry 17, no. 1: 221. 10.1186/s12888-017-1383-2. 28619017 PMC5472954

[brb370752-bib-0007] Cruz, D. 2023. “Childhood Trauma Questionnaire‐Short Form: Evaluation of Factor Structure and Measurement Invariance.” Journal of Child & Adolescent Trauma 16, no. 4: 1099–1108. 10.1007/s40653-023-00556-8. 38045834 PMC10689687

[brb370752-bib-0008] Dannlowski, U. , H. Kugel , F. Huber , et al. 2013. “Childhood Maltreatment Is Associated With an Automatic Negative Emotion Processing Bias in the Amygdala.” Human Brain Mapping 34, no. 11: 2899–2909. 10.1002/hbm.22112. 22696400 PMC6870128

[brb370752-bib-0009] Dannlowski, U. , A. Stuhrmann , V. Beutelmann , et al. 2012. “Limbic Scars: Long‐Term Consequences of Childhood Maltreatment Revealed by Functional and Structural Magnetic Resonance Imaging.” Biological Psychiatry 71, no. 4: 286–293. 10.1016/j.biopsych.2011.10.021. 22112927

[brb370752-bib-0011] de Bles, N. J. , L. E. H. Pütz , N. Rius Ottenheim , et al. 2023. “Childhood Trauma and Anger in Adults With and Without Depressive and Anxiety Disorders.” Acta Psychiatrica Scandinavica 148, no. 3: 288–301. 10.1111/acps.13589. 37430486

[brb370752-bib-0012] di Tella, M. , M. Adenzato , C. Catmur , F. Miti , L. Castelli , and R. B. Ardito . 2020. “The Role of Alexithymia in Social Cognition: Evidence From a Non‐Clinical Population.” Journal of Affective Disorders 273: 482–492. 10.1016/j.jad.2020.05.012. 32560944

[brb370752-bib-0013] Ditzer, J. , E. Y. Wong , R. N. Modi , M. Behnke , J. J. Gross , and A. Talmon . 2023. “Child Maltreatment and Alexithymia: A Meta‐Analytic Review.” Psychological Bulletin 149, no. 5–6: 311–329. 10.1037/bul0000391. 37261746

[brb370752-bib-0014] Divac, N. , M. Prostran , I. Jakovcevski , and N. Cerovac . 2014. “Second‐Generation Antipsychotics and Extrapyramidal Adverse Effects.” BioMed Research International 2014: 656370. 10.1155/2014/656370. 24995318 PMC4065707

[brb370752-bib-0015] Fett, A. K. , W. Viechtbauer , M. D. Dominguez , D. L. Penn , J. van Os , and L. Krabbendam . 2011. “The Relationship Between Neurocognition and Social Cognition With Functional Outcomes in Schizophrenia: A Meta‐Analysis.” Neuroscience and Biobehavioral Reviews 35, no. 3: 573–588. 10.1016/j.neubiorev.2010.07.001. 20620163

[brb370752-bib-0016] Gebreegziabhere, Y. , K. Habatmu , A. Mihretu , M. Cella , and A. Alem . 2022. “Cognitive Impairment in People With Schizophrenia: An Umbrella Review.” European Archives of Psychiatry and Clinical Neuroscience 272, no. 7: 1139–1155. 10.1007/s00406-022-01416-6. 35633394 PMC9508017

[brb370752-bib-0017] Gong, X. , Y.‐X. Huang , Y. Wang , and Y.‐J. Luo . 2011. “Revision of the Chinese Facial Affective Picture System.” Chinese Mental Health Journal 25, no. 1: 40–46.

[brb370752-bib-0018] Harris, A. 2023. “Approach to Schizophrenia.” Internal Medicine Journal 53, no. 4: 473–480. 10.1111/imj.16068. 37070777

[brb370752-bib-0019] Hart, H. , L. Lim , M. A. Mehta , A. Simmons , K. A. H. Mirza , and K. Rubia . 2018. “Altered Fear Processing in Adolescents With a History of Severe Childhood Maltreatment: An fMRI Study.” Psychological Medicine 48, no. 7: 1092–1101. 10.1017/s0033291716003585. 29429419 PMC6088776

[brb370752-bib-0020] Haviland, M. G. , J. L. Sonne , and P. A. Kowert . 2004. “Alexithymia and Psychopathy: Comparison and Application of California Q‐Set Prototypes.” Journal of Personality Assessment 82, no. 3: 306–316. 10.1207/s15327752jpa8203_06. 15151806

[brb370752-bib-0021] Huang, G. , C. Qiu , M. Liao , Q. Gong , L. Liu , and P. Jiang . 2024. “Association of Neuroimaging Measures With Facial Emotional Processing in Healthy Adults: A Task fMRI Study.” Social Cognitive and Affective Neuroscience 19, no. 1: sae076. 10.1093/scan/nsae076. PMC11570540 39420729

[brb370752-bib-0022] Huo, L. , D. Qu , C. Pei , et al. 2023. “Alexithymia in Chronic Schizophrenia and Its Mediating Effect Between Cognitive Deficits and Negative Symptoms.” Schizophrenia Research 261: 275–280. 10.1016/j.schres.2023.10.006. 37866075

[brb370752-bib-0023] Jauhar, S. , M. Johnstone , and P. J. McKenna . 2022. “Schizophrenia.” Lancet 399, no. 10323: 473–486. 10.1016/s0140-6736(21)01730-x. 35093231

[brb370752-bib-0024] Javed, A. , and A. Charles . 2018. “The Importance of Social Cognition in Improving Functional Outcomes in Schizophrenia.” Frontiers in Psychiatry 9: 157. 10.3389/fpsyt.2018.00157. 29740360 PMC5928350

[brb370752-bib-0025] Jiang, W. J. , B. L. Zhong , L. Z. Liu , Y. J. Zhou , X. H. Hu , and Y. Li . 2018. “Reliability and Validity of the Chinese Version of the Childhood Trauma Questionnaire‐Short Form for Inpatients With Schizophrenia.” PLoS One 13, no. 12: e0208779. 10.1371/journal.pone.0208779. 30543649 PMC6292582

[brb370752-bib-0026] Joukamaa, M. , J. Miettunen , P. Kokkonen , et al. 2001. “Psychometric Properties of the Finnish 20‐Item Toronto Alexithymia Scale.” Nordic Journal of Psychiatry 55, no. 2: 123–127. 10.1080/08039480151108561. 11802910

[brb370752-bib-0027] Kadri, N. , M. Agoub , S. El Gnaoui , M. Alami Kh , T. Hergueta , and D. Moussaoui . 2005. “Moroccan Colloquial Arabic Version of the Mini‐International Neuropsychiatric Interview (MINI): Qualitative and Quantitative Validation.” European Psychiatry 20, no. 2: 193–195. 10.1016/j.eurpsy.2004.11.007. 15797707

[brb370752-bib-0028] Lee, S. C. , G. H. Lin , C. L. Shih , et al. 2022. “Error Patterns of Facial Emotion Recognition in Patients With Schizophrenia.” Journal of Affective Disorders 300: 441–448. 10.1016/j.jad.2021.12.130. 34979185

[brb370752-bib-0029] Liu, Y. , D. Zhang , Y. Zhao , S. Tan , and Y. Luo . 2016. “Deficits in Attentional Processing of Fearful Facial Expressions in Schizophrenic Patients.” Scientific Reports 6: 32594. 10.1038/srep32594. 27586404 PMC5009338

[brb370752-bib-0030] Maercker, A. , T. Michael , L. Fehm , E. S. Becker , and J. Margraf . 2004. “Age of Traumatisation as a Predictor of Post‐Traumatic Stress Disorder or Major Depression in Young Women.” British Journal of Psychiatry 184: 482–487. 10.1192/bjp.184.6.482. 15172941

[brb370752-bib-0031] McCrory, E. J. , S. A. De Brito , P. A. Kelly , et al. 2013. “Amygdala Activation in Maltreated Children During Pre‐Attentive Emotional Processing.” British Journal of Psychiatry 202, no. 4: 269–276. 10.1192/bjp.bp.112.116624. 23470285

[brb370752-bib-0032] Montebarocci, O. , P. Surcinelli , N. Rossi , and B. Baldaro . 2011. “Alexithymia, Verbal Ability and Emotion Recognition.” Psychiatric Quarterly 82, no. 3: 245–252. 10.1007/s11126-010-9166-7. 21188637

[brb370752-bib-0033] Pollak, S. D. , M. Messner , D. J. Kistler , and J. F. Cohn . 2009. “Development of Perceptual Expertise in Emotion Recognition.” Cognition 110, no. 2: 242–247. 10.1016/j.cognition.2008.10.010. 19059585 PMC2673797

[brb370752-bib-0034] Read, J. , B. D. Perry , A. Moskowitz , and J. Connolly . 2001. “The Contribution of Early Traumatic Events to Schizophrenia in Some Patients: A Traumagenic Neurodevelopmental Model.” Psychiatry 64, no. 4: 319–345. 10.1521/psyc.64.4.319.18602. 11822210

[brb370752-bib-0035] Rokita, K. I. , M. R. Dauvermann , and G. Donohoe . 2018. “Early Life Experiences and Social Cognition in Major Psychiatric Disorders: A Systematic Review.” European Psychiatry 53: 123–133. 10.1016/j.eurpsy.2018.06.006. 30144982

[brb370752-bib-0036] Sedat, D. , S. Esat , O. Ismail , et al. 2013. “Facial Emotion Recognition in Patients With Violent Schizophrenia.” Schizophrenia Research 144, no. 1–3: 142–145. 10.1016/j.schres.2012.12.015. 23333505

[brb370752-bib-0037] Sedgwick, O. , S. Young , D. Baumeister , B. Greer , M. Das , and V. Kumari . 2017. “Neuropsychology and Emotion Processing in Violent Individuals With Antisocial Personality Disorder or Schizophrenia: The Same or Different? A Systematic Review and Meta‐Analysis.” Australian and New Zealand Journal of Psychiatry 51, no. 12: 1178–1197. 10.1177/0004867417731525. 28992741

[brb370752-bib-0038] Suzuki, A. , L. Poon , V. Kumari , and A. J. Cleare . 2015. “Fear Biases in Emotional Face Processing Following Childhood Trauma as a Marker of Resilience and Vulnerability to Depression.” Child Maltreatment 20, no. 4: 240–250. 10.1177/1077559515600781. 26294753

[brb370752-bib-0039] Tottenham, N. , T. A. Hare , A. Millner , T. Gilhooly , J. D. Zevin , and B. J. Casey . 2011. “Elevated Amygdala Response to Faces Following Early Deprivation.” Developmental Science 14, no. 2: 190–204. 10.1111/j.1467-7687.2010.00971.x. 21399712 PMC3050520

[brb370752-bib-0040] Vaskinn, A. , K. N. Engelstad , A. K. Torgalsbøen , and B. R. Rund . 2021. “Childhood Trauma, Social Cognition and Schizophrenia: Specific Association Between Physical Neglect and Cognitive Theory of Mind in Homicide Offenders.” Psychiatry Research 303: 114093. 10.1016/j.psychres.2021.114093. 34247060

[brb370752-bib-0041] Weiblen, R. , N. Mairon , S. Krach , et al. 2021. “The Influence of Anger on Empathy and Theory of Mind.” PLoS One 16, no. 7: e0255068. 10.1371/journal.pone.0255068. 34324527 PMC8321371

[brb370752-bib-0042] Wells, R. , I. Jacomb , V. Swaminathan , et al. 2020. “The Impact of Childhood Adversity on Cognitive Development in Schizophrenia.” Schizophrenia Bulletin 46, no. 1: 140–153. 10.1093/schbul/sbz033. 31050754 PMC6942153

[brb370752-bib-0043] Wu, B. J. , T. H. Lan , T. M. Hu , S. M. Lee , and J. Y. Liou . 2015. “Validation of a Five‐Factor Model of a Chinese Mandarin Version of the Positive and Negative Syndrome Scale (CMV‐PANSS) in a Sample of 813 Schizophrenia Patients.” Schizophrenia Research 169, no. 1–3: 489–490. 10.1016/j.schres.2015.09.011. 26443481

[brb370752-bib-0044] Xiao, Y. , J. Tian , Y. F. Pan , et al. 2024. “The Prevalence of Alexithymia in Schizophrenia: A Systematic Review and Meta‐Analysis.” Asian Journal of Psychiatry 102: 104280. 10.1016/j.ajp.2024.104280. 39461046

[brb370752-bib-0045] Yehya, A. , S. Ghuloum , Z. Mahfoud , et al. 2016. “Validity and Reliability of the Arabic Version of the Positive and Negative Syndrome Scale.” Psychopathology 49, no. 3: 181–187. 10.1159/000447328. 27475457

[brb370752-bib-0046] Yi, Y. , Y. Huang , R. Jiang , et al. 2023. “The Percentage and Clinical Correlates of Alexithymia in Stable Patients With Schizophrenia.” European Archives of Psychiatry and Clinical Neuroscience 273, no. 3: 679–686. 10.1007/s00406-022-01492-8. 36239818 PMC10085932

[brb370752-bib-0047] Zeng, H. , X. Zhang , W. Wang , et al. 2020. “Maternal Separation With Early Weaning Impairs Neuron‐Glia Integrity: Non‐Invasive Evaluation and Substructure Demonstration.” Scientific Reports 10, no. 1: 19440. 10.1038/s41598-020-76640-y. 33173142 PMC7656452

[brb370752-bib-0048] Zhang, T. Y. , B. Labonté , X. L. Wen , G. Turecki , and M. J. Meaney . 2013. “Epigenetic Mechanisms for the Early Environmental Regulation of Hippocampal Glucocorticoid Receptor Gene Expression in Rodents and Humans.” Neuropsychopharmacology 38, no. 1: 111–123. 10.1038/npp.2012.149. 22968814 PMC3521971

[brb370752-bib-0049] Zhang, X. , M. Dalmaso , L. Castelli , et al. 2021. “Social Attention Across Borders: A Cross‐Cultural Investigation of Gaze Cueing Elicited by Same‐ and Other‐Ethnicity Faces.” British Journal of Psychology 112, no. 3: 741–762. 10.1111/bjop.12476. 33010036

